# The Relevance of Emotional Intelligence in Personnel Selection for High Emotional Labor Jobs

**DOI:** 10.1371/journal.pone.0154432

**Published:** 2016-04-28

**Authors:** Sarah Herpertz, Sophia Nizielski, Michael Hock, Astrid Schütz

**Affiliations:** 1 Department of Psychology, University of Bamberg, Bamberg, Germany; 2 Department of Psychology, Chemnitz University of Technology, Chemnitz, Germany; Universitat Wien, AUSTRIA

## Abstract

Although a large number of studies have pointed to the potential of emotional intelligence (EI) in the context of personnel selection, research in real-life selection contexts is still scarce. The aim of the present study was to examine whether EI would predict Assessment Center (AC) ratings of job-relevant competencies in a sample of applicants for the position of a flight attendant. Applicants’ ability to regulate emotions predicted performance in group exercises. However, there were inconsistent effects of applicants’ ability to understand emotions: Whereas the ability to understand emotions had a positive effect on performance in interview and role play, the effect on performance in group exercises was negative. We suppose that the effect depends on task type and conclude that tests of emotional abilities should be used judiciously in personnel selection procedures.

## Introduction

Although the importance of measuring applicants’ emotional intelligence (EI) in the context of personnel selection is widely acknowledged [1], research is still relatively scarce. To address this gap, the present study assessed applicants’ EI in a real-life selection setting and examined whether facets of EI are related to Assessment Center (AC) ratings of job-relevant competencies.

EI is a research area that has seen some debate. The psychological construct of EI is associated with competing concepts [2]. There are two established scientific concepts of EI: (a) the ability model and (b) mixed and trait models. According to the ability model [3], EI is a form of intelligence that links emotion and cognition. Mayer and Salovey [4] strictly separated ability EI from personality traits. They proposed the four-branch model of EI comprising the abilities to (a) perceive, appraise, and express emotions, (b) use emotions to facilitate thought, (c) understand and analyze emotions, and employ emotional knowledge, and (d) regulate emotions to promote emotional and intellectual growth.

Another related conceptualization of ability EI has been provided by Joseph and Newman [5]. The authors conceptualized ability EI as cascading model, which consists of three abilities: 1) emotion perception, 2) emotion understanding, and 3) emotion regulation. These three abilities form a hierarchy, whereby the ability to perceive emotions facilitates the development of emotion understanding, which in turn enables the development of emotion regulation. In contrast to the model proposed by Mayer and Salovey [4], the branch emotion facilitation is not included due to the conceptual overlap with other EI dimensions and its lack of empirical distinctiveness [6–8]. Meta-analytic data as well as a recent study on the university and cultural specificity across the three branches support the structure of the cascading model of EI [5, 9].

The mixed and trait models concept, such as the Bar-On model of emotional-social intelligence [10], conceptualize EI more broadly [11] and has therefore often been the target of criticism in the scientific community. In contrast to the ability model, which strictly separates EI from personality traits, mixed and trait EI models explicitly combine mental abilities with other constructs [12]. Thus, by definition, several parts are outside of the realm of intelligence [13]. More specifically, mixed models of EI combine mental abilities, traditional personality traits, and dispositions, such as motivation and optimism (for a review, see [14]), whereas the trait EI model covers personality traits that are associated with affect. In other words, trait models of EI mix emotion-related self-perceptions (e.g., self-perceived ability to regulate emotions) with dispositions (e.g., self-esteem, happiness [15]).

The most prevalent approaches to EI are performance-based and self-report measures. To classify EI research, Ashkanasy and Daus [16] identified a three-stream approach. Based on this model, EI research can be categorized in 1) the ability model assessed by performance-based test, or 2) the ability model assessed by self-report, and 3) expanded models (e.g., mixed models) assessed by self-report. In this study we focused on the ability model of EI as the concept is much more narrowly defined than mixed and trait EI: Ability EI comprises specific mental abilities in processing emotional information and is conceptually separate from personality.

Impression management and socially desirable responding is a particular problem within the context of personnel selection. In general, self-ratings can be distorted by self-serving biases such as self-enhancement or impression management [17]. Lievens, Klehe, and Libbrecht [18], for example, found that applicants’ scores on self-reported EI are significantly higher and have less variance than incumbents’ scores. Second, and specific to EI, people may have limited self-knowledge concerning their emotional abilities. By contrast, performance-based tests like the Mayer-Salovey-Caruso Emotional Intelligence Test (MSCEIT [19]) can be considered more valid than self-reports, because this approach is resistant to faking good [20] and unrelated to cognitive biases such as overestimating one’s own skills [19]. Thus, we decided to use the performance-based approach to ability EI instead of self-report.

The MSCEIT [19] is a widely used problem-based measure that explicitly assesses the abilities to perceive, understand, and regulate emotions. A systematic association between gender and all MSCEIT scales has repeatedly been found [21]. For the German version of the MSCEIT correlations were between *r* = .08 (emotion understanding) and *r* = .14 (emotion regulation), with females scoring consistently higher than males.

Previous research indicates that not everybody benefits from high EI with regard to one’s own job performance. In a meta-analytical study, Joseph and Newman [5] found that the positive relation between EI and job performance held only for people in jobs which require emotional labor and demand employees to show emotions that are in accordance with the display rules of the organization even if their private feelings diverge from these. By contrast, they found no or even a negative relation between EI and job performance for people working in low emotional labor jobs. Accordingly, it seems plausible that personnel selection procedures for high emotional labor jobs may be improved by measuring applicants’ EI. Thus, the study presented below was aimed at examining whether and how EI would predict Assessment Center (AC) ratings for people applying for a high emotional labor job.

Jobs with high emotional labor are primarily to be found in the service sector [22]. A prototypical high emotional labor job is the role of a flight attendant [23–25]. For example, regardless of their own discomfort, flight attendants have to express calmness and composure [23], and sometimes they are expected to suppress their own feelings of frustration in the face of angry or disruptive passengers to maintain a professional appearance [26]. Very few studies have examined the relevance of emotional abilities in real-life selection settings. Most of these studies were based on mixed/trait EI conceptualizations and used samples of medical school applicants [27]. In a pilot study, Carrothers and colleagues [27] tested mixed EI (comprising maturity, compassion, morality, sociability, and calm disposition) in applicants for a medical school. As expected, applicants’ EI was significantly related to their personal and interpersonal competencies as rated by the interviewers. Moreover, successful applicants had higher EI scores than those who were not accepted, even though EI scores were not used in the decision-making process. We use a similar approach to test the relevance of ability EI in the selection procedure for flight attendants.

### The Present Study

To address the issues mentioned above, we conducted a study with a sample of applicants for a position as a flight attendant. More specifically, we tested whether and how applicants’ ability EI predicts ratings of job-relevant competencies during the selection procedure. Because of relatively low construct validity of the ability to use emotions [5, 28], we assessed only three of the four facets in the Mayer and Salovey [4] model of ability EI, namely the abilities to perceive, understand, and regulate emotions.

The role of flight attendant includes a variety of tasks and successful performance requires coping with various demands. In this study, we focused on emotional job demands that could be simulated in an Assessment Center. The job-relevant competencies required to deal with these tasks were interpersonal skills, cooperation and teamwork, customer service orientation, realistic self-evaluation, and stress-resistance as elaborated below.

In line with other social occupations that are characterized by frequent interactions and are sometimes even conflict-prone, interpersonal skills are considered to be essential [29]. Similar to Engelberg and Sjöberg [30], we assume that EI constitutes a foundation for interpersonal skills that supports effective social interactions and relationships. Furthermore, a flight attendant does not only interact with passengers, but is part of a closely collaborating team. Thus, cooperation and teamwork are further demands. Service is a key component of the flight attendant role. This is why customer service orientation is considered an additional demand. In order to improve oneself in the flight attendant role, it is also important to acquire a realistic idea of oneself and one’s own job performance which in turn requires self-reflection and the ability to take criticism. Finally, flight attendants face a lot of stressors at work, when they, for example, have to deal with complaining passengers. Thus, stress-resistance can be considered another job-relevant competency.

We expect that emotionally intelligent applicants will be more likely to possess these job-relevant competencies which should help them to perform successfully. Thus, we hypothesize that applicants’ emotional abilities (i.e., the abilities to perceive, understand, and regulate emotions) are positively related to AC ratings of these job-relevant competencies.

## Method

### Ethical Statement

The study was approved by the ethics committee of the Institute of Psychology, University of Bamberg. Participants provided written informed consent. In the case of participants under 18 years, the airline collected written consent of the guardians.

### Participants and Procedure

We analyzed the data of 193 individuals who had applied for flight attendant positions to a large German airline—138 of whom were later accepted. Women comprised 78.8% of the sample. Ages ranged from 17 to 52 years (*M* = 25.88, *SD* = 7.13).

If they passed height requirements and a telephone interview assessing English language skills and job motivation they were accepted to the airline’s AC. In addition, participants completed a computerized test of EI.

### Measures

#### Emotional intelligence

The German version of the Mayer-Salovey-Caruso Emotional Intelligence Test (MSCEIT [21]), a performance-based test, was used to assess ability EI. The MSCEIT represents Mayer and Salovey’s [4] model of emotional intelligence. Consistent with our focus on the ability to perceive emotions in others, the ability to understand emotions, and the ability to regulate emotions, we used only an abbreviated form of the MSCEIT. In order to measure the ability to perceive emotions in others (henceforth, simply called *the ability to perceive emotions*), applicants worked on the faces task, for which they had to correctly identify emotions in four photographs of faces. Answers were given on a 5-point rating scale ranging from 1 (*no/not at all*) to 5 (*extreme/very strong*). We did not use the pictures task of the perceiving emotions branch as the faces task has a high split-half reliability on its own.

The ability to understand emotions was assessed by two tasks. Applicants were asked to indicate how emotions change over time and how blends of emotions result in complex feelings. Participants responded by choosing one out of five answer options. The ability to regulate emotions was assessed by asking participants to rate the effectiveness of different strategies to manage their own emotions and the emotions of others on a 5-point scale ranging from 1 (*very ineffective*) to 5 (*very effective*). Applicants’ scores were calculated using the consensus scoring method. The test has split-half reliability coefficients of .86 for the faces task, and .72 and .73 for the branches understanding and regulating emotions, respectively [21].

#### Job-relevant competencies and characteristics

Applicants participated in the airline’s standard AC for flight attendants, which included simulated selection scenarios in order to assess applicants’ job-relevant competencies and characteristics. Each applicant was observed by a psychologist during a group exercise, which was designed to represent typical job demands such as equipping a serving trolley that fits for a particular purpose. The psychologist was not involved in the present study and blind with respect to the hypotheses. He or she rated each applicant with respect to (a) interpersonal skills in group interaction and (b) cooperation and teamwork. Ratings were made on a Likert-type scale ranging from 1 (*low*) to 5 (*high*).

Another psychologist (not involved in the present study either and blind with respect to the hypotheses) conducted a structured interview with each applicant. During the interview procedure, the applicants also participated in a role play, in which the psychologist took the passenger’s role and the applicant took the flight attendant’s role. The psychologist rated each applicant with respect to (a) motivation and realistic job view, (b) professional conduct and physical appearance, (c) interpersonal skills in dyadic interaction, (d) customer service orientation, (e) realistic self-evaluation, and (f) stress-resistance. Behavioral anchors were used to facilitate ratings of all job-relevant competencies and characteristics (examples of behavioral anchors are available from the authors on request).

In this study, we are interested in whether applicants with high emotional abilities are more likely to meet the emotional demands of the flight attendant role than their less emotionally intelligent competitors. Some of the competencies and characteristics (e.g., physical appearance) that were assessed in the AC were not related to emotional labor and were not included in the present study. The present study focused on the following job-relevant competencies: interpersonal skills in group interaction, cooperation and teamwork (assessed in the AC group exercise); interpersonal skills in dyadic interaction, customer-service orientation, realistic self-evaluation, and stress-resistance (assessed in the interview and the AC role play).

#### Data analysis

Data analysis using the free statistical software R version 3.2.2 [31] comprised three steps: (a) descriptive analyses, (b) principal component analysis, and (c) path analysis. We used principal component analysis of AC ratings of applicants’ job-relevant competencies to avoid potential redundancies in the AC data and to gain less interdependent variables. By using component scores instead of the original ratings, we could also assess and increase the reliability of our dependent measures. To examine the relation between applicants’ emotional abilities and AC performance, we conducted a path analysis.

## Results

### Descriptive Statistics, Intercorrelations, and Comparisons of Means

Descriptive statistics are reported in Table 1 and intercorrelations are reported in Table 2. In highlighting some of the findings here we would like to point out that male and female applicants differed in their ability to perceive emotions: The Welch’s t-test showed that females have higher scores than males [*t*(51) = -2.20, *p* = .033; female: *M* = 109.66, *SD* = 11.62; male: *M* = 103.54, *SD* = 16.79; Cohen’s *d* = 0.48]. Furthermore, applicants’ perceived stress-resistance increased with age (*r* = .16, *p* = .025).

**Table 1:** Descriptive Statistics.

Variable	M	SD
**MSCEIT**		
Perceiving emotions	108.36	13.08
Understanding emotions	102.96	14.91
Regulating emotions	101.75	15.28
**Job-relevant competencies assessed in the interview**		
Motivation and realistic job view	3.24	0.64
Professional conduct and physical appearance	3.13	0.65
Interpersonal skills in dyadic interaction	3.05	0.68
Stress-resistance	3.24	0.52
**Job-relevant competencies assessed in the role play**		
Customer service orientation	3.09	0.72
Realistic self-evaluation	3.11	0.52
**Job-relevant competencies assessed in the group exercise**		
Interpersonal skills in group interaction	3.03	0.56
Cooperation and teamwork	3.15	0.55

*N* = 193. MSCEIT = Mayer-Salovey-Caruso Emotional Intelligence Test.

**Table 2:** Pearson Product-Moment Correlations.

Variable	1	2	3	4	5	6	7	8	9	10	11
**MSCEIT**											
1 Perceiving emotions		.27**	.34**	.05	.11	.11	-.04	.02	.03	.10	.04
2 Understanding emotions			.25**	-.03	.01	.09	.08	.09	.17*	-.14	-.13
3 Regulating emotions				.08	.05	.12	-.02	.09	.04	.12	.10
**Job-relevant competencies assessed in the interview**											
4 Motivation and realistic job view					.52**	.57**	.26**	.50**	.51**	.33**	.31**
5 Professional conduct and physical appearance						.71**	.36**	.52**	.42**	.49**	.42**
6 Interpersonal skills in dyadic interaction							.42**	.63**	.52**	.40**	.36**
7 Stress-resistance								.39**	.28**	.09	.07
**Job-relevant competencies assessed in the role play**											
8 Customer service orientation									.44**	.32**	.26**
9 Realistic self-evaluation										.20**	.21**
**Job-relevant competencies assessed in the group exercise**											
10 Interpersonal skills in group interaction											.69**
11 Cooperation and teamwork											

*N* = 193. MSCEIT = Mayer-Salovey-Caruso Emotional Intelligence Test.

*p < .05.

**p < .01, two-tailed.

### Principle Component Analysis of the AC Ratings of Applicants’ Job-Relevant Competencies

As shown in Table 2, the AC ratings of applicants’ job-relevant competencies were highly correlated, pointing to potential redundancies in the data. We therefore examined the dimensionality of the AC ratings by conducting a principal component analysis of these ratings using the R package psych 1.5.8 [32]. Parallel analysis results based on the principal components of the rating data [33] suggested two components with eigenvalues that exceeded those of the random variables. After a Varimax rotation of the loadings, these components explained 38% and 31% of the variance, respectively. As shown in Table 3, the rotation yielded a clear structure. Ratings of applicants’ job-relevant competencies based on their performance in the interview and the role play loaded highly only on the first principal component (henceforth simply called *interview and role play ratings of job-relevant competencies*) and ratings of applicants’ job-relevant competencies based on their performance in the group exercise loaded highly only on the second component (simply called *group exercise ratings of job-relevant competencies*). The reliabilities (Cronbach’s α) of the sum scores of the *interview and role play* and the *group exercise* ratings were .77 and .81, respectively, and thus satisfactory. Moreover, these estimates closely corresponded to more accurate reliability estimates from a confirmatory independent cluster model (ω [34]), in which the items of the two groups were assigned to different factors (ω was .79 for the interview and role play factor, and .81 for the group exercise factor [35]). The resulting component scores (which were nearly identical to the sum scores, *r*s > .97) were used as dependent variables in the subsequent path analysis.

**Table 3:** Varimax Rotated Two-Components Structure of the AC Ratings of Job-Relevant Competencies.

Job-relevant competencies	*a*_*1*_	*a*_*2*_	*h*^*2*^
**Assessed in the interview**			
Interpersonal skills in dyadic interaction	.78	.36	.74
Stress-resistance	.73	-.11	.55
**Assessed in the role play**			
Customer service orientation	.77	.24	.66
Realistic self-evaluation	.71	.15	.52
**Assessed in the group exercise**			
Interpersonal skills in group interaction	.15	.90	.82
Cooperation and teamwork	.11	.90	.82

*N* = 193. *a*_*1*_ = loadings on the first principal component; *a*_*2*_ = loadings on the second principal component; *h*^*2*^ = communality.

### Path Analysis

To investigate whether applicants’ emotional abilities had positive effects on AC performance, we conducted a path analysis. The path model test was conducted with the R-package lavaan [36] using the Satorra-Bentler mean-adjusted maximum-likelihood estimation method, which yields a standard maximum likelihood estimation of the model parameters with robust standard errors and a Satorra-Bentler scaled test statistic [37].

We tested a model that included the three EI facets as predictors and the two principal components (i.e., interview and role play ratings of job-relevant competencies and group exercise ratings of job-relevant competencies) as outcomes (see S1 Fig). We included gender (coded 0 for male and 1 for female participants) as an additional predictor in the model because male and female applicants differed significantly in the ability to perceive emotions, as described above. As stress-resistance increased with age, we examined age as another additional predictor (age was coded with 0 for participants below the median of the age distribution and 1 for participants above the median; *Mdn* = 24.0). Model fit was very good with a nonsignificant chi-square statistic (χ^2^ = 9.10, *df* = 8, *p* = .33), CFI = .98, TLI = .95, RMSEA = .03, SRMR = .05.

Partially supporting our hypothesis, there was a significant positive effect of the ability to understand emotions on interview and role play performance (*β* = .19, *p* = .02) as well as a significant positive effect of the ability to regulate emotions on group exercise performance (*β* = .15, *p* = .034). By contrast, the effect of the ability to understand emotions on group exercise performance was negative (*β* = -.24, *p* = .002).

## Discussion

The central aim of our study was to examine whether emotionally intelligent applicants would be more likely to meet the demands of a job requiring high emotional labor than their competitors. Thus, we examined whether emotionally intelligent applicants would receive higher AC ratings than their less emotionally intelligent counterparts. We found a positive effect of the ability to understand emotions on interview and role play performance and a positive effect of the ability to regulate emotions on group exercise performance. These findings were expected and indicate that the ability to perceive and regulate emotions helps persons to deal with demanding situations in the AC. An interesting but unexpected finding was the negative effect of the ability to understand emotions on group exercise performance.

The inconsistent effects of applicants’ ability to understand emotions point to the relevance of the situation (interview and role play vs. group exercise) for EI effects: The group exercise was performed under time pressure and therefore represents a more stressful situation than the interview and the role play. Moreover, interview and role-play are dyadic situations whereas there are multiple interaction partners in the group exercise. The ability to understand emotions is the “most cognitively saturated” [38] EI facet and may be beneficial in situations that require planning and contemplation, but could actually be harmful in stressful, unpredictable situations, in which spontaneous reactions are required. In these situations emotion regulation ability should be especially relevant, as was shown in our data.

Interpreting one’s own emotional cues and the cues of others might prohibit spontaneous behavior and demand cognitive resources that are needed to act adaptively in such a group situation. We believe that applicants with high ability to understand emotions might have been under increased cognitive load, as they were analyzing the situation instead of acting spontaneously. Consequently, they might have received low ratings of interpersonal skills in group interaction, cooperation, and teamwork in the group exercise.

By contrast, the interview and the role play were more predictable situations comprising dyadic interactions. Thus, applicants high in the ability to understand emotions might outperform others who are less able to understand emotions because they have the cognitive resources available to adhere to existing norms and act upon behavioral scripts. This strategy seems to be adaptive in interview situations. In a similar vein, Sieverding [39] found that individuals who tried to suppress emotions during the interview according to conventional display rules, received higher competence ratings than so-called nonsuppressors (i.e., those individuals who did not try to hide or suppress emotions).

Finally, we found that applicants’ perceived stress-resistance increased with age. This finding is in accordance with a previous review of a lifespan perspective on emotion regulation and stress in the workplace [40]: Older employees perceived daily hassles less negatively [41] and tended to report reduced affective reactivity to stressful events [42]. We assume that older applicants handle the potentially stressful selection situation of the AC more successfully and thus received higher ratings of stress-resistance than their younger competitors.

### Limitations and Directions for Future Research

Some limitations of this study should be noted. First, based on the work of Hochschild [26] who describes the emotional demands of the flight attendant role in an impressive way, we took it for granted that the role of flight attendant is associated with high emotional labor. However, we recommend to assess emotional labor as an additional control variable in future research. It is not clear whether the emotional demands of current flight attendants are similar to those more than 30 years ago or whether the demands vary between attendants and could account for interindividual differences.

A critical aspect of the selection process was that only one psychologist provided ratings in each situation. Although such less-than-optimal approaches are not uncommon in real-life selection settings [43], in future studies, at least two judges should be employed in each rating situation. This procedure would additionally allow for computing the inter-rater reliability of the AC ratings.

We used the MSCEIT [21] to assess applicants’ EI. Some subscales (i.e., understanding and regulating emotions) of this measure have only moderate reliabilities, and this in turn reduces power to observe associations with the predictor and outcome variables. With a more reliable measure, larger effects may have emerged. Furthermore, because the data were cross-sectional in nature, we cannot infer whether EI predicts long-term job success of the accepted applicants. We thus recommend that future research applies a longitudinal approach and examines the mental health and job performances of accepted applicants over a period of time. In addition, the incremental validity of EI above and beyond other selection variables (e.g., cognitive intelligence and conscientiousness) needs to be established more thoroughly.

### Practical Implications

Focusing on the abilities to perceive and regulate emotions seems to be useful for selecting individuals applying for high emotional labor jobs. Our results suggest that applicants’ emotional abilities impact their behavior in selection situations. This behavior, in turn, predicts ratings of job-relevant competencies. Personnel selection may be improved by integrating a performance-based measure of the ability to regulate emotions because applicants’ scores on regulating emotions seem to be indicative of their ability to work as team members and to meet the corresponding demands (e.g., cooperation) of a high emotional labor job. When focusing on the ability to understand emotions, a differential approach may be needed as suggested by our results regarding dyadic and group selection situations—a cognitive focus on understanding emotions may help in some situations but harm in others. Even though future studies are needed to replicate the present results, including measures of applicants’ EI in an evidence based fashion in the selection process may improve traditional personnel selection procedures for jobs requiring high emotional labor.

## Supporting Information

S1 Fig Path model with standardized coefficients. Gender was coded with 0 for male and 1 for female participants. Age was coded with 0 for participants below the median of the age distribution and 1 for participants above the median. *N* = 193. ^†^*p* < .10. *p < .05. **p < .01.
(TIFF)

S1 File Data file_160410.sav. (SAV)
