# Supplementary figures and images for: The Relevance of Emotional Intelligence in Personnel Selection for High Emotional Labor Jobs
Source: PLoS One. 2016 Apr 28;11(4):e0154432. doi: 10.1371/journal.pone.0154432 (PMC4849674; doi:10.1371/journal.pone.0154432)

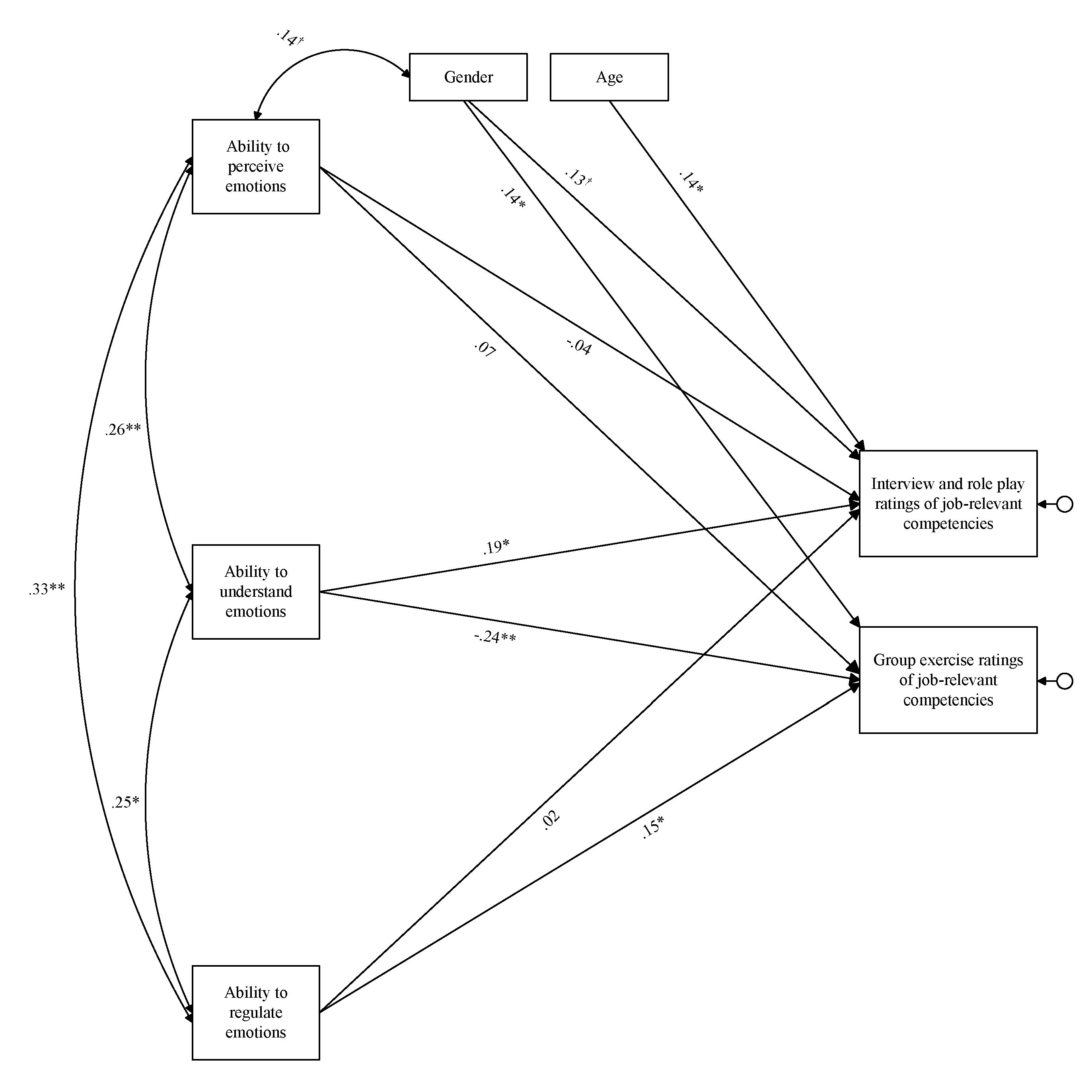

Supplement: S1 Fig — Gender was coded with 0 for male and 1 for female participants. Age was coded with 0 for participants below the median of the age distribution and 1 for participants above the median. N = 193. †p < .10. *p < .05. **p < .01. (TIFF) [file pone.0154432.s001.tiff]
